# Pulmonary capillary reserve and exercise capacity at high altitude in healthy humans

**DOI:** 10.1007/s00421-015-3299-1

**Published:** 2015-11-27

**Authors:** Bryan J. Taylor, Kirsten E. Coffman, Douglas T. Summerfield, Amine N. Issa, Alex J. Kasak, Bruce D. Johnson

**Affiliations:** Division of Cardiovascular Diseases, Department of Internal Medicine, Mayo Clinic, Rochester, USA; School of Sport and Health Science, College of Life and Environmental Sciences, University of Exeter, St. Luke’s Campus, Heavitree Road, EX1 2LU Exeter, UK; Division of Aerospace Medicine, Mayo Clinic, Rochester, USA; School of Kinesiology, University of Minnesota, Minneapolis, USA

**Keywords:** Lung diffusing capacity, Maximal oxygen consumption, Pulmonary artery systolic pressure, Sea level

## Abstract

**Purpose:**

We determined whether well-acclimatized humans have a reserve to recruit pulmonary capillaries in response to exercise at high altitude.

**Methods:**

At sea level, lung diffusing capacity for carbon monoxide (DLCO), alveolar-capillary membrane conductance (Dm_CO_), and pulmonary capillary blood volume (*V*_c_) were measured at rest before maximal oxygen consumption ($\dot{V}{\text{O}}_{2\hbox{max} }$) was determined in seven adults. Then, DLCO, Dm_CO_ and *V*_c_ were measured pre- and post-exhaustive incremental exercise at 5150 m after ~40 days of acclimatization.

**Results:**

Immediately after exercise at high altitude, there was an increase in group mean Dm_CO_ (14 ± 10 %, *P* = 0.040) with no pre- to post-exercise change in group mean DLCO (46.9 ± 5.8 vs. 50.6 ± 9.6 ml/min/mmHg, *P* = 0.213) or *V*_c_ (151 ± 28 vs. 158 ± 37 ml, *P* = 0.693). There was, however, a ~20 % increase in DLCO from pre- to post-exercise at high altitude (51.2 ± 0.2 vs. 61.1 ± 0.2 ml/min/mmHg) with a concomitant increase in Dm_CO_ (123 ± 2 vs. 156 ± 4 ml/min/mmHg) and *V*_c_ (157 ± 3 vs. 180 ± 8 ml) in 2 of the 7 participants. There was a significant positive relationship between the decrease in $\dot{V}{\text{O}}_{2\hbox{max} }$ from sea level to high altitude and the change in DLCO and lung diffusing capacity for nitric oxide (DLNO) from rest to end-exercise at high altitude.

**Conclusion:**

These data suggest that recruitment of the pulmonary capillaries in response to exercise at high altitude is limited in most well-acclimatized humans but that any such a reserve may be associated with better exercise capacity.

## Introduction

In healthy humans, the elevation in cardiac output and pulmonary perfusion pressure during exercise cause a marked expansion of the highly compliant pulmonary capillary network (La Gerche et al. 2010; Lalande et al. 2012; Stokes et al. 1981; Tamhane et al. 2001; Taylor et al. 2014). Indeed, the increase in lung diffusing capacity for carbon monoxide (DLCO) and nitric oxide (DLNO), alveolar-capillary membrane conductance (Dm_CO_) and pulmonary capillary blood volume (*V*_c_) associated with whole body exercise is indicative of both recruitment of previously closed pulmonary capillaries and distension of the perfused pulmonary vessels (Johnson et al. 1960; Pavelescu et al. 2013; Stokes et al. 1981; Tamhane et al. 2001). At sea level, DLCO, DLNO, Dm_CO_ and *V*_c_ increase linearly with increasing workload with no evidence of a plateau in these variables throughout exhaustive exercise (Hsia et al. 1995; Johnson et al. 1960; Tamhane et al. 2001; Taylor et al. 2014; Zavorsky et al. 2004). This suggests that recruitment and distension of the pulmonary capillaries do not become limited even during heavy- to maximal exercise in healthy humans. That the expansion of the pulmonary capillaries appears not to reach a maximum during exercise likely serves to allow an increase in the alveolar-capillary surface area for effective gas exchange whilst minimizing the exercise-induced increase in pulmonary arterial pressure (PAP), pulmonary vascular resistance (PVR) and right ventricular afterload, all of which have been associated with greater exercise aerobic capacity at sea level (La Gerche et al. 2010; Lalande et al. 2012; Pavelescu et al. 2013).

Exposure to airway hypoxia and/or high altitude also elicits pulmonary capillary recruitment due to an increase in pulmonary perfusion pressure secondary to hypoxic pulmonary vasoconstriction (Brimioulle et al. 1996; de Bisschop et al. 2012; Taylor et al. 2011). At rest, it is probable that the increase in alveolar-capillary surface area associated with recruitment of the pulmonary capillaries improves ventilation–perfusion matching and thus better preserves systemic oxygen levels in humans at high altitude. However, in the setting of ongoing hypoxic pulmonary vasoconstriction, it is possible that the associated recruitment and distension of the pulmonary capillaries may limit or encroach upon the reserve for expansion of the pulmonary vasculature in response to whole body exercise at high altitude. That is, we considered it possible that exposure to hypobaric hypoxia may elicit maximal, or near maximal, recruitment and distension of the pulmonary vasculature such that no reserve remains for further expansion of the pulmonary capillaries in response to exercise at high altitude. Any such limitation in pulmonary capillary recruitment and distension during exercise at high altitude would likely be associated with an inability to adequately increase gas exchange surface area along with an excessive rise in PAP and PVR relative to the metabolic demand of exercise, which in turn would be expected to significantly impair exercise capacity (La Gerche et al. 2010; Lalande et al. 2012; Naeije et al. 2010; Pavelescu et al. 2013).

It has been shown previously that a large pulmonary vascular reserve, defined as low pulmonary vascular resistance in combination with elevated lung diffusing capacity, is associated with better aerobic exercise capacity at high altitude (Pavelescu et al. 2013). However, we are unaware of any previous study that has assessed whether well-acclimatized healthy humans maintain a reserve to recruit and distend the pulmonary vasculature in response to exercise at high altitude. Accordingly, the aim of the present study was to determine (1) whether well-acclimatized humans have a reserve to further recruit and distend the pulmonary capillaries in response to exercise at high altitude, and (2) whether such reserve is related to aerobic exercise capacity at high altitude. Experimentally, we reasoned that individuals with a reserve to recruit and/or distend the pulmonary capillaries in response to exercise at high altitude would exhibit a large pre- to post-exercise increase in DLCO, DLNO, Dm_CO_ and *V*_c_. Moreover, we expected that individuals in whom a pulmonary vascular reserve was evidenced at high altitude would also exhibit better maintenance of exercise capacity (i.e., a higher $\dot{V}{\text{O}}_{2\hbox{max} }$) at high altitude compared to individuals who exhibited no such pulmonary vascular reserve.

## Methods

### Subjects

Seven healthy nonsmoking North American adults (5 male, 2 female) with no history of cardiorespiratory or metabolic disease participated in the study (mean ± SD; age = 35 ± 10 years, stature = 177 ± 11 cm, body mass = 74.6 ± 12.0 kg). The subjects were physically active (≥30 min physical activity/day, ≥5 days/week; self-reported) and had normal forced vital capacity (FVC = 120 ± 11 % of predicted), forced expiratory volume in 1 s (FEV_1_ = 108 ± 7 % of predicted), FEV_1_/FVC ratio (96 ± 6 % of predicted) and maximal mid-expiratory flow (MMEF = 114 ± 10 % of predicted) at sea level. Each participant gave written informed consent after being provided a detailed description of the study requirements. The experimental procedures were approved by the Mayo Clinic Institutional Review Board and were performed in accordance with the ethical standards of the Declaration of Helsinki. All study participants were prohibited from prophylactic administration of any medication to aid altitude acclimatization (e.g., sildenafil, acetazolamide). Moreover, no subject required emergent pharmaceutical treatment (e.g., dexamethasone) for high altitude illness. During the trek to high altitude, two subjects were given an acute course (2 days) of a broad spectrum antibiotic for the treatment of a suspected gastro-intestinal infection.

### Experimental procedures

**Fig. 1:**
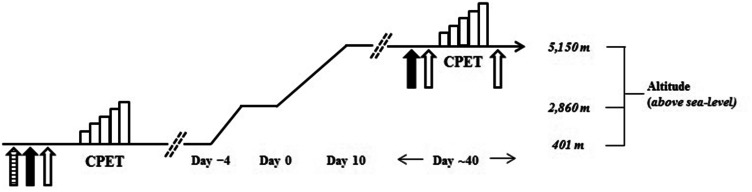
Schematic depiction of the experimental procedures used in this study. At sea level, pulmonary function (*stripped up arrow*), systolic pulmonary arterial pressure (sPAP) (*solid up arrow*) and lung diffusing capacity for carbon monoxide and nitric oxide (DLCO and DLNO), alveolar-capillary membrane conductance (Dm_CO_) and pulmonary capillary blood volume (*V*_c_) (open up arrow) we measured at rest before subject performed a maximal incremental cardiopulmonary exercise test (CPET) on a motorized treadmill. Within 2 weeks of baseline sea level testing, each subject traveled to Kathmandu, Nepal (elevation 1400 m) before being transported by airplane to Lukla, Nepal (elevation 2860 m). From Lukla, the participants completed an 8–10 day hike at progressively increasing altitudes to reach Mount Everest Base Camp. After a further ~30 days of acclimatization to high altitude, sPAP was measured at rest (*solid up arrow*) before DLCO, DLNO, Dm_CO_ and *V*_c_ were assessed before and immediately after a CPET to volitional exhaustion at Mount Everest Base Camp. Due to the logistical constraints of performing exercise at high altitude, CPET at Base camp was conducted by having subjects perform 1 min of step exercise at a rate of 60 steps per min, before the work rate was increased by 30 steps per min every 1 min until they were unable to maintain the required step rate and/or reached volitional exhaustion; the step height was set at 20 cm and the participants maintained the required step rate by following a metronome

At sea level (Rochester, MN, USA; elevation 401 m), pulmonary function was assessed using a spirometer according to standard procedures (Miller et al. 2005) before pulmonary artery systolic pressure (sPAP) (via echocardiography) and lung diffusing capacity for carbon monoxide and nitric oxide (DLCO and DLNO) were measured at rest (Fig. 1). Next, the cardiorespiratory responses to maximal incremental treadmill exercise were assessed in each participant as described previously (Bruce et al. 1963) (Fig. 1). Within 2 weeks of completion of the sea level measurements, each subject traveled to Kathmandu, Nepal (elevation 1400 m) before being transported by airplane to Lukla, Nepal (elevation 2860 m). From Lukla, the participants completed an 8–10 day hike at progressively increasing altitudes to reach Mount Everest Base Camp. After a further ~30 days of acclimatization to high altitude (group mean maximum elevation reached 6385 ± 917 m, range 5150–7200 m), sPAP was measured at rest before DLCO, DLNO, alveolar-capillary membrane conductance (Dm_CO_) and pulmonary capillary blood volume (*V*_c_) were assessed before and immediately after incremental step exercise performed to volitional exhaustion at Mount Everest Base Camp (Fig. 1).

### Pulmonary artery pressure

sPAP was estimated from the peak velocity of tricuspid regurgitation (TR) using a modified Bernoulli equation as described previously (Taylor et al. 2011; Yock and Popp 1984). With the participants in the left lateral supine position, the TR jet was located using 2D-color Doppler echocardiography (SonoSite Edge, FUJIFILM SonoSite Inc., Bothell, WA). To determine the maximal velocity of the TR jet, the continuous wave sampler was positioned within and parallel to the regurgitation jet and sPAP was computed as 4TR^2^ added to an assumed right atrial pressure of 5 mmHg.

### Lung diffusing capacity

With subjects in the sitting position, DLCO and DLNO were assessed by simultaneously measuring the disappearance of CO and NO via a rapid single breath technique using an automated device for performing gas calibrations, extemporaneous mixing of gases and calculations (Hyp’air Compact, Medisoft, Dinant, Belgium) (de Bisschop et al. 2012; Pavelescu et al. 2013). For each single breath maneuver, the participants were instructed to breathe normally on environmental air for 4–5 breaths before exhaling slowly and completely down to residual volume (RV). Once at RV, the participants were switched to an inspiratory reservoir filled with 2600 ppm CO, 40 ppm NO, 8 % He, 21 % O_2_ and N_2_ balance, and told to inspire rapidly and fully to total lung capacity before holding their breath for 4 s. After the breath hold, the participants then exhaled steadily and swiftly back to RV. The first 0.9 L of the expired gas was discarded to ensure dead-space wash out with the next 0.9 L of the expirate collected for subsequent analysis. The single breath maneuver was performed in duplicate before (<10 min) and after maximal exercise at high altitude. The first post-exercise single breath maneuver was performed within 20 s of end-exercise with the second performed 4 mins later, according to current guidelines (Macintyre et al. 2005). Importantly, pilot data from our laboratory have suggested that transient exposure to 40 ppm NO during the assessment of lung diffusing capacity has no discernible effect on systolic pulmonary artery pressure in humans exposed to normobaric hypoxia (FIO_2_ 12.5 %) for ~17 h (sPAP: 32.7 ± 5.9 pre-DLCO/DLNO assessment vs. 31.6 ± 6.4 mmHg post-DLCO/DLNO assessment, *n* = 5). Thus, it appears that the transient administration NO gas associated with our assessment of DLNO and DLNO does not alter pulmonary vascular tone such that our measures of DLCO, Dm_CO_ and *V*_c_ would be affected.

Following the assessment of lung diffusing capacity, Dm_CO_ and *V*_c_ were computed as described previously (de Bisschop et al. 2012; Glenet et al. 2007; Pavelescu et al. 2013). Based on the molecular weight and solubility of CO and NO, the coefficient relating DLNO to Dm_CO_ was set at 1.97 (Aguilaniu et al. 2008) such that Dm_CO_ was calculated as the measured DLNO/1.97. Then, to solve the Roughton and Forster equation (Roughton and Forster 1957), 1/$\ominus_{\text{CO}}$ was calculated using an equation proposed by Forster expressing the blood conductance of CO (i.e. $\ominus_{\text{CO}}$) as a function of capillary PO_2_ (Forster 1987):

$$1/\ominus_{\text{CO}} = { 1}. 3 { } + \, 0.00 4 1 { } \times {\text{ PcapO}}_{ 2}$$

where PcapO_2_ is the capillary pressure of O_2_, estimated as alveolar PO_2_ − $\dot{V}{\text{O}}_{2}$/(DLCO × 1.23) with partial pressures in mmHg, $\dot{V}{\text{O}}_{2}$ in ml/min, and DLCO in ml/min/mmHg. Based on the measured barometric pressure (~400 mmHg) and the expired fraction of O_2_, the calculated alveolar PO_2_ at Everest Base Camp ranged from 55 to 58 mmHg. $\dot{V}{\text{O}}_{2}$ was calculated using the mass balance of O_2_ between inspiration and expiration during the single breath maneuver and DLCO × 1.23 was used as a surrogate for DLO_2_ (Forster, 1987). Using this equation, PcapO_2_ was calculated as 120 ± 7 mmHg and 53 ± 5 mmHg at sea level and Everest Base Camp, respectively; these values are similar to those recently reported under similar conditions (de Bisschop et al. 2012). Venous blood was sampled for hemoglobin (Hb) concentration and *V*_c_ was corrected accordingly for standard concentrations of Hb in men (14.6 g/dl) and women (13.4 g/dl) (Macintyre et al., 2005) as measured *V*_c_ × (standard Hb concentration/measured Hb concentration). To allow comparison between DLCO measured at sea level and high altitude, DLCO at Everest Base Camp was recalculated using the Dm_CO_ and *V*_c_ values computed at high altitude and the sea level (i.e., normoxic) $\ominus_{\text{CO}}$ as follows (Pavelescu et al. 2013):

$$1/{\text{DLCO}}_{\text{ALT}} = { 1}/{\text{Dm}}_{\text{COALT}} + { 1}/\ominus_{{{\text{CO}}_{\text{SL}} }} \cdot V_{{{\text{c}}_{\text{ALT}} }}$$

where, Dm_COALT_ and *V*_cALT_ are the alveolar-capillary membrane conductance and pulmonary capillary blood volume, respectively, calculated at Everest Base Camp using the hypoxic $\ominus_{\text{CO}}$ and $\ominus_{{{\text{CO}}_{\text{SL}} }}$ is the $\ominus_{\text{CO}}$ at sea level (i.e., the normoxic $\ominus_{\text{CO}}$).

### Cardiopulmonary exercise test at high altitude

Following 2 min of quiet rest, the participants performed 1 min of step exercise at a rate of 60 steps per min, before the work rate was increased by 30 steps per min every 1 min until they were unable to maintain the required step rate and/or reached volitional exhaustion. The step height was set at 20 cm and the participants maintained the required step rate by following a metronome. Ventilatory and pulmonary gas exchange indices, including minute ventilation ($\dot{V}_{\text{E}}$), oxygen consumption ($\dot{V}{\text{O}}_{2}$) and carbon dioxide production ($\dot{V}{\text{CO}}_{2}$), were measured breath-by-breath using a system with integrated pneumotachometer (VO_2000_ Metabolic Measurement System, MGC Diagnostics, St. Paul, MN) and averaged over the last 20 s of each minute during exercise. Similarly, heart rate (HR) and estimated arterial oxygen saturation (SaO_2_) were assessed beat-by-beat during exercise using a pulse oximeter (NONIN PureSAT Model 3150 Oximeter, NONIN Medical Inc., Plymouth, MN) with finger sensor, and averaged over the last 20 s of each minute during exercise. From these parameters, derived variables including $\dot{V}_{\text{E}}$/$\dot{V}{\text{CO}}_{2}$ ratio, respiratory exchange ratio (RER) and oxygen pulse ($\dot{V}{\text{O}}_{2}$/HR) were calculated and averaged as described above. Ratings of perceived exertion (dyspnea and whole body) were obtained at rest, at 1 min of exercise, and every 1 min thereafter using Borg’s 6–20 scale.

### Statistical analyses

The within-subject coefficient of variation (CV) for DLCO and DLNO was used to estimate the sample size required to detect meaningful changes in DLCO and DLNO from before to after the cardiopulmonary exercise test at high altitude (approximately double the CV) given a statistical power of 0.8 and an alpha level of 0.05 (Hopkins et al. 1999). Based on a CV of 4.78 % for DLCO and 4.94 % for DLNO (calculated in our laboratory), respectively, a sample size of 7 allowed us to detect a 7.5 % change in DLCO and an 8.0 % change in DLNO. The CV was determined using the method error of the measurement. Repeated measures ANOVA was used to compare absolute measures of lung diffusing capacity and related variables (DLCO, DLNO, Dm_CO_ and *V*_c_) across time (sea level at rest vs. altitude at rest before exercise vs. altitude immediately after exercise). When significant main effects were shown, planned pair-wise comparisons were made with the Bonferroni method. Paired samples *t* test was used to compare the cardiorespiratory (e.g., $\dot{V}_{\text{E}}$, $\dot{V}{\text{O}}_{2}$, HR, SaO_2_ and derived variables) and perceptual (RPE dyspnea and RPE whole body) responses to the final minute of maximal incremental exercise at sea level vs. at high altitude. Paired samples *t* test was also used to compare resting sPAP at sea level vs. at high altitude. Pearson’s product-moment correlation coefficient (*r*) was computed to assess (1) the relationship between the absolute change (∆) in DLCO and DLNO from before to after exercise at high altitude and the absolute change (∆) in $\dot{V}{\text{O}}_{2\hbox{max} }$ from sea level to high altitude, and (2) the relationship between pulmonary artery systolic pressure and $\dot{V}{\text{O}}_{2\hbox{max} }$ measured at sea level and at high altitude. The acceptable type I error was set at *P* < 0.05. Where appropriate, data are expressed as group mean ± SD. Statistical analyses were performed using SPSS version 12.0 for Windows (SPSS, Chicago, IL).

## Results

### General effects of high altitude

Overall, the participants acclimatized well to high altitude and reported only mild, transient degrees of headache and fatigue/weakness during the 5 days preceding experimentation at Everest Base Camp. On the morning of the experimentation day at Everest Base Camp, group mean Lake Louise score (2 ± 1) was not positive for the presence of acute mountain sickness. From sea level to the day of experimentation at Everest Base Camp, there was a decrease in resting SaO_2_ (97 ± 1 vs. 84 ± 5 %, *P* < 0.001) and an increase in resting HR (69 ± 14 vs. 83 ± 9 beats/min, *P* = 0.035), hemoglobin concentration (15.4 ± 1.4 vs. 17.8 ± 1.4 g/dL, *P* = 0.007) and sPAP (19.5 ± 4.5 vs. 29.2 ± 3.2 mmHg, *P* = 0.002).

### Lung diffusing capacity

#### Lung diffusing capacity at rest: sea level vs. high altitude

Resting measures of DLCO, Dm_CO_ and *V*_c_ at sea level and on the day of experimentation at Everest Base Camp are shown in Table 1. From sea level to high altitude, there was a significant increase in group mean DLCO (10 ± 4 %, *P* < 0.001) and Dm_CO_ (18 ± 11 %, *P* = 0.007). By contrast, group mean *V*_c_ at rest was not different at high altitude compared to sea level (151 ± 28 vs. 139 ± 34 ml, *P* = 0.667) (Table 1). There was no change in resting DLNO/DLCO and resting Dm_CO_/*V*_c_ from sea level to high altitude (Table 1).

**Table 1:** Resting lung diffusion variables at sea level and after acclimation to high altitude

	Sea level	High altitude	*P* value
DLCO, ml/min/mmHg	42.8 ± 6.2	46.9 ± 5.8	<0.001
DLNO, ml/min/mmHg	178 ± 26	208 ± 26	0.007
DLNO/DLCO	4.19 ± 0.56	4.46 ± 0.43	0.594
Dm_CO_, ml/min/mmHg	90 ± 13	106 ± 13	0.007
*V*_c_, ml	139 ± 34	151 ± 28	0.667
Dm_CO_/*V*_c_	0.69 ± 0.18	0.71 ± 0.13	0.731

Values are group mean ± SD for 7 subjects (2 female). Measures at high altitude (5150 m) made after ~40 days of high altitude acclimatization

*DLCO* lung diffusing capacity for carbon monoxide, *DLNO* lung diffusing capacity for nitric oxide, *Dm*_*CO*_ alveolar-capillary membrane conductance, *V*_c_ pulmonary capillary blood volume

#### Effect of exercise at high altitude on lung diffusing capacity

**Fig. 2:**
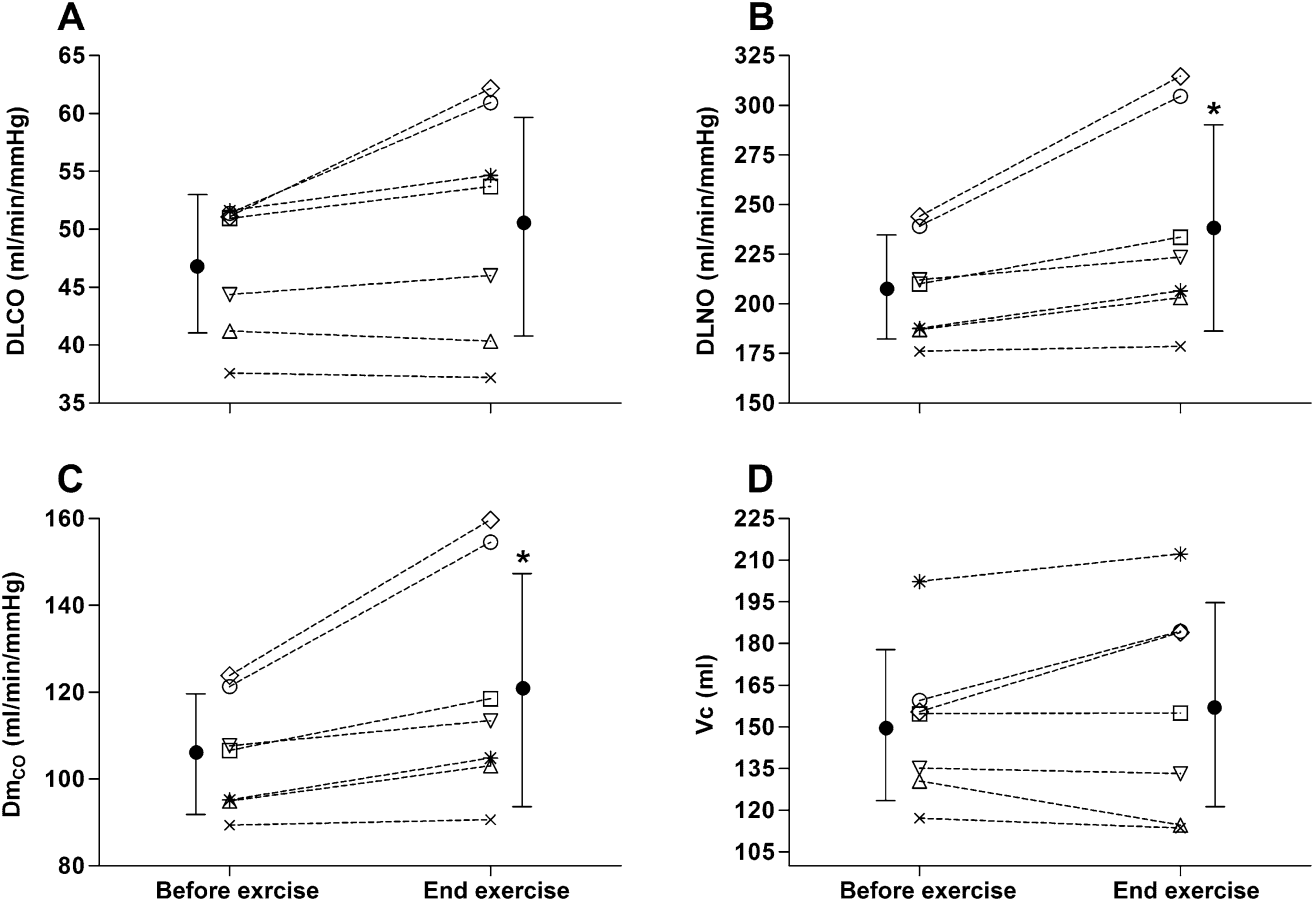
Individual subject (*dashed lines*) and group mean (*solid* *circles*) changes in lung diffusing capacity for carbon monoxide (DLCO, *Panel A*), lung diffusing capacity for nitric oxide (DLNO, *Panel B*), alveolar-capillary membrane conductance (Dm_CO_, *Panel C*) and pulmonary capillary blood volume (*V*_c_, *Panel D*) from pre- to post-exercise at high altitude. The female subjects are represented as the *open square* and the *open up triangle*. *P < 0.05, group mean value significantly different after vs. before exercise

Individual participant and group mean changes in DLCO, Dm_CO_ and *V*_c_ from before to after exercise at high altitude are shown in Fig. 2. Immediately after exercise, there was a small but significant increase in group mean Dm_CO_ (14 ± 10 %, *P* = 0.040) with no pre- to post-exercise change in group mean DLCO (46.9 ± 5.8 vs. 50.6 ± 9.6 ml/min/mmHg, *P* = 0.213) or *V*_c_ (151 ± 28 vs. 158 ± 37 ml, *P* = 0.693) (Fig. 2). There was, however, a ~20 % increase in DLCO from before to after exercise at high altitude (51.2 ± 0.2 vs. 61.1 ± 0.2 ml/min/mmHg) with a concomitant increase in Dm_CO_ (123 ± 2 vs. 156 ± 4 ml/min/mmHg) and *V*_c_ (157 ± 3 vs. 180 ± 8 ml) in 2 of the 7 participants (denoted by the open circle and open diamond in Fig. 1). In the remaining 5 subjects, there was either a minimal increase (subjects denoted by the star and the open square in Fig. 1) or no change in DLCO, Dm_CO_ and *V*_c_ from before to after exercise at high altitude (Fig. 1).

### Cardiorespiratory responses to exercise: sea level vs. high altitude

The cardiorespiratory and perceptual responses to the final minute of maximal incremental exercise at sea level and at high altitude are shown in Table 2. Compared to sea level, altitude exposure was associated with a decrease in peak $\dot{V}{\text{O}}_{2}$ (−20 ± 7 %, *P* = 0.002), peak $\dot{V}_{\text{E}}$ (−29 ± 9 %, *P* = 0.001), peak HR (−19 ± 4 %, *P* < 0.001), O_2_pulse (−8 ± 6 %, *P* = 0.014) and SaO_2_ (−20 ± 5 %, *P* < 0.001) at end-exercise. RER, $\dot{V}_{\text{E}}$/$\dot{V}{\text{CO}}_{2}$, O_2_pulse and perceptions of dyspnea and whole body discomfort were not different at end-exercise at high altitude compared to at sea level (Table 2).

**Table 2:** Responses to the final minute of the exhaustive cardiopulmonary exercise test (CPET) at sea level and after acclimation to high altitude

	Sea level	High altitude	*P* value
${\text{V}}_{{{\text{O}}_{ 2} }}$, ml/kg/min	48.7 ± 5.2	39.0 ± 2.9 (80 ± 5)	0.002
V_E_, l/min	135 ± 19	95 ± 24 (70 ± 5)	0.001
RER	1.18 ± 0.08	1.12 ± 0.06 (96 ± 3)	0.128
V_E_/${\text{V}}_{{{\text{CO}}_{ 2} }}$	32.9 ± 2.2	35.3 ± 2.2	0.408
HR, beats/min	183 ± 19	148 ± 16 (81 ± 4)	<0.001
O_2_pulse, ml/beat	19.3 ± 3.7	18.0 ± 4.5	0.014
SaO_2_, %	96 ± 1	77 ± 5	<0.001
RPE dyspnea, CR10	9.7 ± 0.7	9.5 ± 1.0 (98 ± 2)	0.392
RPE whole body, Borg 6-20	18.8 ± 0.9	18.3 ± 1.0 (97 ± 4)	0.415

Values are group mean ± SD for 7 subjects (2 female). Values in parentheses represent percent of sea level values. Measures at high altitude (5150 m) made after ~ 40 days of high altitude acclimation

${\text{V}}_{{{\text{O}}_{ 2} }}$ oxygen consumption, *V*_*E*_ minute ventilation, *RER* respiratory exchange ratio, *HR* heart rate, *O*_*2*_ *pulse* oxygen pulse (oxygen consumption/HR), *SaO*_*2*_ arterial oxygen saturation, *RPE* rating of perceived exertion

### Correlations

**Fig. 3:**
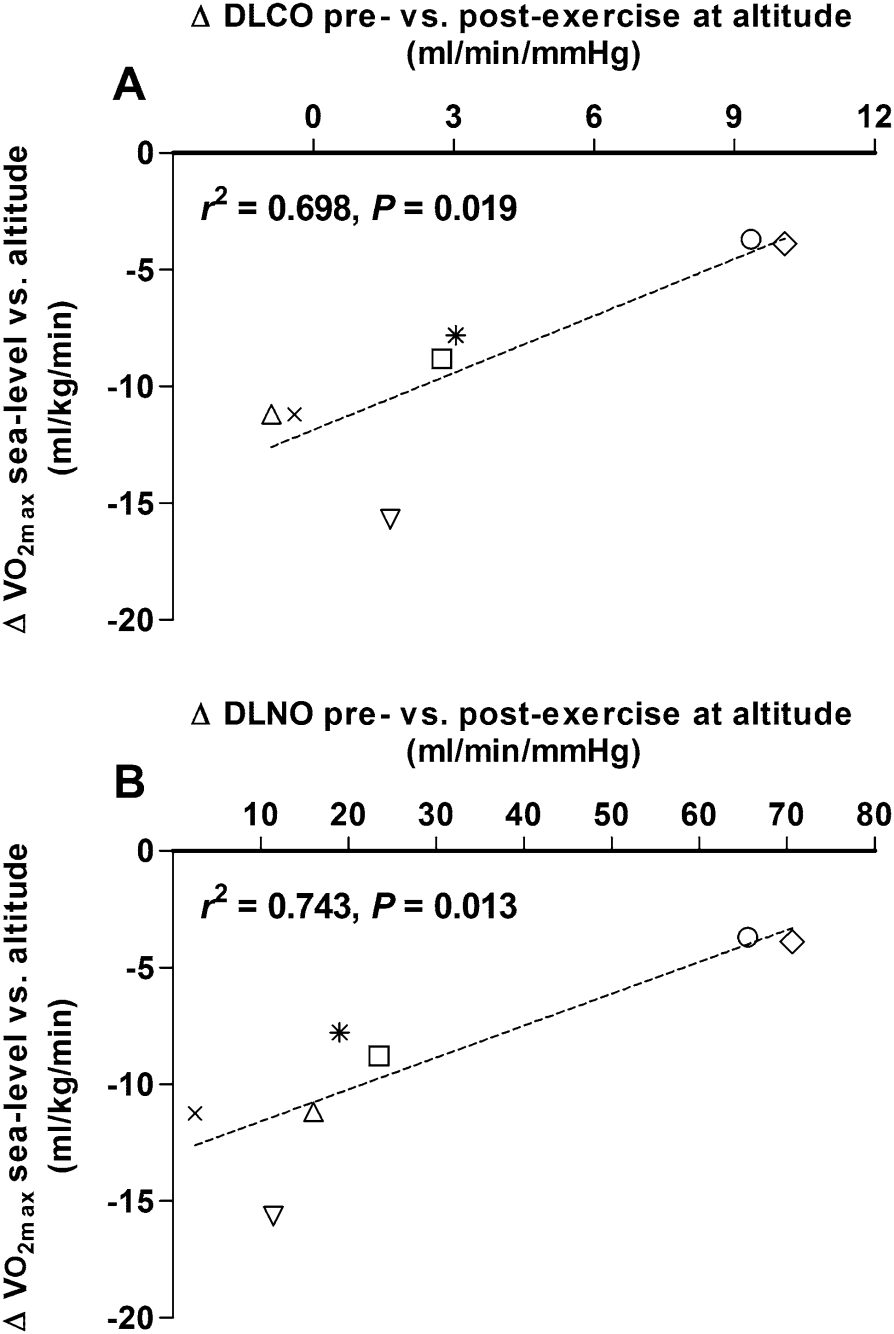
Scatter plots showing relationships between the individual subject maximal oxygen consumption ($\dot{V}{\text{O}}_{2\hbox{max} }$) at high altitude and the pre- to post-exercise change in lung diffusing capacity for carbon monoxide (DLCO, *Panel A*) and lung diffusing capacity for nitric oxide (DLNO, *Panel B*) at high altitude

There was a significant positive relationship between the magnitude of the decrease in $\dot{V}{\text{O}}_{2\hbox{max} }$ from sea level to high altitude and the change in DLCO and DLNO from rest to end-exercise at high altitude (*r*^2^ = 0.698, *P* = 0.019; and *r*^2^ = 0.743, *P* = 0.013, respectively) (Fig. 3). In addition, there was a significant negative relationship between resting sPAP and $\dot{V}{\text{O}}_{2\hbox{max} }$ at high altitude (*r*^2^ = − 0.517, *P* = 0.041) but not at sea level (*r*^*2*^ = 0.141, *P* = 0.464).

## Discussion

### Main findings

The main finding of the present study was that exhaustive incremental exercise at high altitude caused a small but significant increase in group mean lung diffusing capacity for nitric oxide (DLNO) and alveolar-capillary membrane conductance (Dm_CO_), but no change in lung diffusing capacity for carbon monoxide (DLCO) or pulmonary capillary blood volume (*V*_c_). There was, however, a large (~20 %) increase in DLCO from before to immediately after exercise at high altitude with a concomitant increase Dm_CO_ and *V*_c_ in two of the seven subjects studied, with minimal or no increase in these parameters in the remaining participants. A large pre- to post-exercise increase in DLCO and its related variables was considered to be ≥15 % (Horita et al. 2015). Interestingly, there was a significant positive relationship between the magnitude of the decrease in $\dot{V}{\text{O}}_{2\hbox{max} }$ from sea level to high altitude and the change in DLCO and DLNO from rest to end-exercise at high altitude. In combination, these findings suggest that (1) recruitment of the pulmonary capillaries in response to exercise at high altitude is limited in most well-acclimatized humans, and (2) there is perhaps a very small number of individuals who do maintain a reserve to recruit and distend the pulmonary capillaries in response to exercise in this environment, and that the presence of such a reserve is associated with better maintained aerobic exercise capacity at high altitude.

### Role of pulmonary capillary reserve in the determination of exercise capacity at high altitude

It is well known that exercise capacity is substantially limited at high altitude, even in well-acclimatized healthy humans (Calbet et al. 2003; Fulco et al. 1998). While the mechanisms that underpin the decline in exercise capacity at high altitude are multifactorial, it has been reported that a persistent reduction in maximal cardiac output along with a preferential redistribution of blood flow and thus oxygen delivery to non-exercising tissues play a critical role in the failure to recover $\dot{V}{\text{O}}_{2\hbox{max} }$ in acclimatized humans, despite normalization of arterial oxygen content (Calbet et al. 2003; Fulco et al. 1998; Wagner 2000). It is becoming increasingly clear, however, that exercise capacity at high altitude is also related to pulmonary vascular function and alterations in pulmonary vascular pressure and resistance (de Bisschop et al. 2012; Naeije et al. 2010; Pavelescu et al. 2013). In a recent retrospective review of data collected during four separate high altitude expeditions, Pavelescu et al. (2013) found that $\dot{V}{\text{O}}_{2\hbox{max} }$ was negatively related to resting pulmonary artery pressure but positively related to resting lung diffusing capacity for nitric oxide at high altitude as well as at sea level. Moreover, the authors reported that the high altitude related decrease in $\dot{V}{\text{O}}_{2\hbox{max} }$ was inversely related to the increase in resting PVR observed at high altitude (Pavelescu et al. 2013). In addition, a number of previous studies have demonstrated that pharmacologic reduction of pulmonary artery pressure and pulmonary vascular resistance secondary to dilation of the pulmonary vascular resistance vessels is associated with an improvement in exercise capacity and an increase in the membrane component of lung diffusion in humans exposed to hypoxia and/or high altitude (de Bisschop et al. 2012; Faoro et al. 2009; Faoro et al. 2007; Ghofrani et al. 2004; Naeije et al. 2010). For example, Naeije et al. (2010) reported that administration of sitaxsentan, a selective endothelin-A receptor blocker, decreased PVR with a concomitant partial restoration in $\dot{V}{\text{O}}_{2\hbox{max} }$ in subjects exposed to both acute normobaric hypoxia and chronic hypobaric hypoxia. Taken together, the aforementioned findings imply that the presence of a less distensible pulmonary vascular network in combination with the development of hypoxia-mediated pulmonary hypertension contribute substantially to the limitation in aerobic exercise capacity in healthy humans at high altitude. Although the mechanisms by which elevated pulmonary vascular pressures and resistance facilitate a persistent reduction in $\dot{V}{\text{O}}_{2\hbox{max} }$ in this setting are yet to be fully elucidated, it is likely that the increase in PAP and PVR, and the consequent increase in right ventricular afterload, act to limit right ventricular outflow and pulmonary vascular perfusion with a subsequent under-perfusion of well-ventilated alveoli (V/$\dot{\text{Q}}$ mismatching) and a reduction in O_2_ delivery to the working muscles.

It is well known that exposure to airway hypoxia and/or high altitude elicits recruitment of the pulmonary capillaries due to an increase pulmonary perfusion pressure secondary to hypoxic pulmonary vasoconstriction (Brimioulle et al. 1996; de Bisschop et al. 2012; Taylor et al. 2011). In the present study, we theorized that this underlying hypoxia-mediated recruitment and distension of the pulmonary capillaries may limit or encroach upon an individual’s reserve to further expand the pulmonary capillary network in response to exercise at high altitude, and that any such limitation would be a source of diminished exercise capacity in this environment via an impairment in the increase in alveolar-capillary surface area for effective gas exchange and a heightened exercise-induced increase in PAP and PVR. We found that ~40 days of exposure to high altitude was associated with substantial hypoxic pulmonary vasoconstriction and recruitment of the pulmonary capillaries, as evidenced by a significant increase in sPAP (~56 %), DLCO (~10 %) and Dm_CO_ (~18 %) relative to sea level values. In addition, we found that only 2 of the 7 well acclimated healthy humans studied maintained a reserve to further recruit and distend the pulmonary capillaries in response to exercise at high altitude, as evidenced by a substantial pre- to immediately post-exercise increase in DLCO (~20 %), Dm_CO_ (~28 %) and *V*_c_ (~15 %) (Fig. 1). Interestingly, there was a significant positive relationship between the magnitude of the decrease in $\dot{V}{\text{O}}_{2\hbox{max} }$ from sea level to high altitude and the change in DLCO and DLNO from rest to end-exercise at high altitude (Fig. 3). In combination, these data suggest that encroachment upon or lack of a reserve to expand the pulmonary vasculature during exercise may be a significant source of exercise limitation at high altitude even in well-acclimatized individuals, perhaps via a limitation in effective surface area for pulmonary gas exchange and/or an excessive rise in pulmonary vascular pressures and RV afterload.

### Why do some but not all humans maintain a pulmonary capillary reserve at high altitude?

The underlying physiological reason(s) that underpin why pulmonary capillary expansion is largely limited during exercise in most well-acclimatized humans, with only a small number of individuals (2 of 7 in the present study) exhibiting evidence for a reserve to recruit and distend the pulmonary capillaries in response to exercise in this environment, are unknown and cannot be elucidated from the findings of the present study. However, while somewhat speculative, one possible explanation is that lung size is related to the cross-sectional area of the pulmonary vasculature. That is, individuals with larger lungs likely also have a larger pulmonary capillary network and, therefore, are able to maintain a reserve to recruit and/or distend the pulmonary capillaries in response to exercise at high altitude in spite of the underlying pulmonary vascular recruitment associated with hypoxic pulmonary vasoconstriction. In support of this postulation, the two individuals who were able to markedly increase DLCO, Dm_CO_ and *V*_c_ during exercise at high altitude in the present study both had a FVC (6.3 L and 6.2 L) that was greater than the group mean (5.5 L including females, 5.8 L excluding females). A second potential explanation relates to the highly variable degree of pulmonary vasoreactivity in response to hypoxia commonly observed across individuals. Likely contributors to this variation are alterations in iron status, chemosensitivity, estrogen and polymorphisms in several hypoxia sensitive genes, including the serotonin transporter (5-HTT) gene (Austin et al. 2009; Olson et al. 2007; Smith et al. 2008). Theoretically, an abnormally large or excessive rise in pulmonary vascular pressure in response to hypoxic exposure may also be associated with a larger degree of pulmonary vascular recruitment on exposure to high altitude, thus limiting the reserve for further expansion of the pulmonary vasculature in response to exercise. Presently, while we do not have any evidence that directly relates pulmonary vascular pressures to exercise-induced alterations in pulmonary capillary recruitment (i.e., changes in lung diffusing capacity) at high altitude, we did find that $\dot{V}{\text{O}}_{2\hbox{max} }$ was related to baseline pulmonary arterial pressure as well as the change in lung diffusion variables during exercise at high altitude.

### Technical considerations

#### Correlation data: sample size

One potential limitation of the present study is that the somewhat small cohort of subjects studied likely increased the potential of a type I error. Specifically, it is plausible that the clustering of our data into two relatively distinct groups could be, at least in part, contributing to the significant linear relationship we have demonstrated between the change in $\dot{V}{\text{O}}_{2\hbox{max} }$ from sea level to high altitude and the change in DLCO and DLNO in response to exercise at high altitude (see Fig. 3). Despite this concern, the present findings are remarkably similar to several other recent observations. For example, de Bisschop et al. (de Bisschop et al. 2012) demonstrated a significant positive relationship between $\dot{V}{\text{O}}_{2\hbox{max} }$ and resting measures of DLCO and DLNO, at both sea level and high altitude, in a cohort of 22 healthy adults. Similarly, in a review of data from 64 healthy subjects across four different medical expeditions to high altitude, Pavelescu et al. (2013) reported that $\dot{V}{\text{O}}_{2\hbox{max} }$ was positively related to resting measures of DLCO and DLNO, and negatively related to mean pulmonary arterial pressure, again at sea level and at high altitude. Somewhat in agreement with the aforementioned findings, in the present study we found a significant positive relationship between the magnitude of change in $\dot{V}{\text{O}}_{2\hbox{max} }$ from sea level to high altitude and the change in DLCO and DLNO from before to after exercise at high altitude (see Fig. 3). Accordingly, while we concede that the small sample size used in this investigation may have increased the likelihood of a type I error, we believe that the striking similarities between the present findings and the findings reported by others who studied larger subject cohorts provides substantial support to our data. Thus, we are confident in our conclusion that the presence of reserve to recruit and distend the pulmonary capillaries in response to exercise at high altitude is associated with better maintained aerobic exercise capacity in this environment.

#### Specific conductance for NO

Previously, the blood conductance for nitric oxide ($\ominus_{\text{NO}}$) has been assumed to be either infinite (de Bisschop et al. 2012; Pavelescu et al. 2013) or finite (Martinot et al. 2013) with a value of 4.5 ml/min/mmHg calculated in vivo (Borland et al. 2010). However, although the presence of a defined $\ominus_{\text{NO}}$ value is an intriguing issue, its use in the calculation of Dm_CO_ and *V*_c_ remains debated and somewhat controversial. Indeed, while it has been estimated that 37 % of the resistance to NO uptake lies in the 1/$\ominus_{\text{NO}}$·*V*_c_ component (Borland et al. 2010, 2014), it has been argued that application of this figure in the whole body human must be treated with caution as its calculation involved exchange transfusion in dogs, substituting bovine Hb-glutamer 200 for whole blood (Hughes and van der Lee 2013). Additionally, it has also been estimated that DLNO would not need to be adjusted unless the hemoglobin concentration is <8 g/dl (Borland et al. 2010). Thus, it can be postulated that if the overall binding or mass transfer of NO to hemoglobin is not affected by a substantial lowering of Hb concentration (until lower than 8 g/dl), then the resistance proposed to be provided by the red blood cell to NO (i.e. $\ominus_{\text{NO}}$) would not have any major physiological effect on the measurement of alveolar-capillary membrane conductance. Accordingly, there is currently no consensus on the application of the assumption of a finite value for $\ominus_{\text{NO}}$ in the calculation of Dm_CO_ and *V*_c_ in humans. Furthermore, application of a finite $\ominus_{\text{NO}}$ of 4.5 ml/min/mmHg to the data presented in this study resulted in group mean Dm_CO_ values of 143 ± 35, 218 ± 51 and 286 ± 104 ml/min/mmHg at sea level, high altitude pre-exercise and high altitude post-exercise, respectively. These Dm_CO_ values appear excessively large and do not compare favorably to values previously reported in whole body humans (e.g., de Bisschop et al. 2012). As such, in the present manuscript we felt that it was most correct and prudent to calculate our Dm_CO_ and *V*_c_ based on the assumption that $\ominus_{\text{NO}}$ is infinite.

#### Exercise modality and potential recovery of lung diffusing capacity after exercise at high altitude

A potential concern is that the step-based cardiopulmonary exercise test at high altitude did not elicit a sufficient metabolic demand to facilitate pulmonary vascular recruitment, if indeed a reserve for such recruitment remained. However, at end-exercise at high altitude $\dot{V}{\text{O}}_{2}$, $\dot{V}_{\text{E}}$ and HR reached 80 ± 5, 70 ± 5 and 81 ± 4 % of sea level maximum (Table 2); these values are very much in agreement with the typically reported reduction in exercise capacity and percent attainable of sea level values (e.g., de Bisschop et al. 2012; Pavelescu et al. 2013). In addition, group mean RPE dyspnoea and RPE whole body discomfort were remarkably similar at end-exercise at high altitude compared to at sea level (Table 2). Based on the aforementioned considerations, we believe that the stress induced by our step exercise protocol at high altitude was appropriate and sufficient enough to serve as a stimulus for additional pulmonary vascular recruitment, if such a reserve for recruitment remained.

An additional concern is that there may have been some recovery of any exercise-induced pulmonary vascular recruitment and distension during the time between exercise termination and our post-exercise measures of lung diffusing capacity at high altitude. Any such recovery may have allowed DLCO, DLNO, Dm_CO_ and *V*_c_ to return towards pre-exercise values prior to our post-exercise assessment of lung diffusing capacity, potentially causing us to underestimate or even miss any exercise-induced expansion of the pulmonary vascular bed at high altitude. Despite this concern, in the present study the first post-exercise single breath assessment of lung diffusing capacity was performed immediately after exercise termination (within 20 s) with the second performed 4 mins later, according to current guidelines (Macintyre et al. 2005). Importantly, group mean DLCO taken from each single breath measurement was 51.3 ± 9.8 vs. 49.8 ± 10.1 ml/min/mmHg immediately post- vs. 4 min post-exercise, respectively. Similarly, group mean DLNO was 237 ± 53 vs. 239 ± 51 ml/min/mmHg immediately post- vs. 4 min post-exercise, respectively. Thus, it appears that there was very little, if any, recovery in our measures of DLCO and DLNO during the time taken between exercise termination and the completion of our post-exercise assessment of lung diffusion capacity.

## Conclusion

In conclusion, pulmonary capillary recruitment and distension in response to exercise at high altitude is largely limited in most well acclimatized humans, with only two of the seven participants in the present study appearing to maintain a reserve to expand the pulmonary vasculature in response to exercise in this environment, as evidenced by a substantial increase in lung diffusing capacity for carbon monoxide and its component parts alveolar-capillary membrane conductance and pulmonary capillary blood volume. Additionally, the presence of such a reserve may be associated with better maintenance of aerobic exercise capacity at high altitude.

## Abbreviations

CPET: Cardiopulmonary exercise test
DLCO: Lung diffusing capacity for carbon monoxide
DLNO: Lung diffusing capacity for nitric oxide
Dm_CO_: Alveolar-capillary membrane conductance
PAP: Pulmonary arterial pressure
PVR: Pulmonary vascular resistance
RER: Respiratory exchange ratio
RPE: Rating of perceived exertion
sPAP: Systolic pulmonary arterial pressure
*V*_c_: Pulmonary capillary blood volume
$$\dot{V}_{\text{E}}$$: Minute ventilation
$$\dot{V}{\text{O}}_{2}$$: Oxygen consumption
$\dot{V}{\text{CO}}_{2}$: Carbon dioxide production
